# Supplementary material for: Prevalence of intestinal parasitic infections and genetic differentiation of Strongyloides stercoralis among migrant workers from Myanmar, Lao PDR and Cambodia in northeastern Thailand
Source: PLoS One. 2022 Dec 30;17(12):e0279754. doi: 10.1371/journal.pone.0279754 (PMC9803317; doi:10.1371/journal.pone.0279754)
Supplement: S1 Table — (PDF) [file pone.0279754.s001.pdf]

S1 Table Details of individuals found to be infected with *S. stercoralis* using APC.

No	Cases	Gender	Age	Current residence	Years of living in Thailand (TH)	History of using drugs for helminth treatment before entry into TH	History of using drugs for helminth treatment while in TH	Year of most recent visit to home country
1	Cambodia 1	Female	36	Workers' dormitory	3	No	No	2019
2	Cambodia 2	Male	41	Workers' dormitory	6	No	No	No
3	Cambodia 3	Male	36	Workers' dormitory	5	No	No	2018
4	Lao PDR 1	Male	21	Workers' dormitory	7	No	No	2019
5	Lao PDR 2	Male	43	Workers' dormitory	7	No	No	2019
6	Lao PDR 3	Male	56	Workers' dormitory	5	No	No	2018
7	Myanmar 1	Male	30	Workers' dormitory	13	No	No	2017
8	Myanmar 2	Male	32	Workers' dormitory	3	No	No	No
