# Supplementary figures and images for: Prevalence of intestinal parasitic infections and genetic differentiation of Strongyloides stercoralis among migrant workers from Myanmar, Lao PDR and Cambodia in northeastern Thailand
Source: PLoS One. 2022 Dec 30;17(12):e0279754. doi: 10.1371/journal.pone.0279754 (PMC9803317; doi:10.1371/journal.pone.0279754)

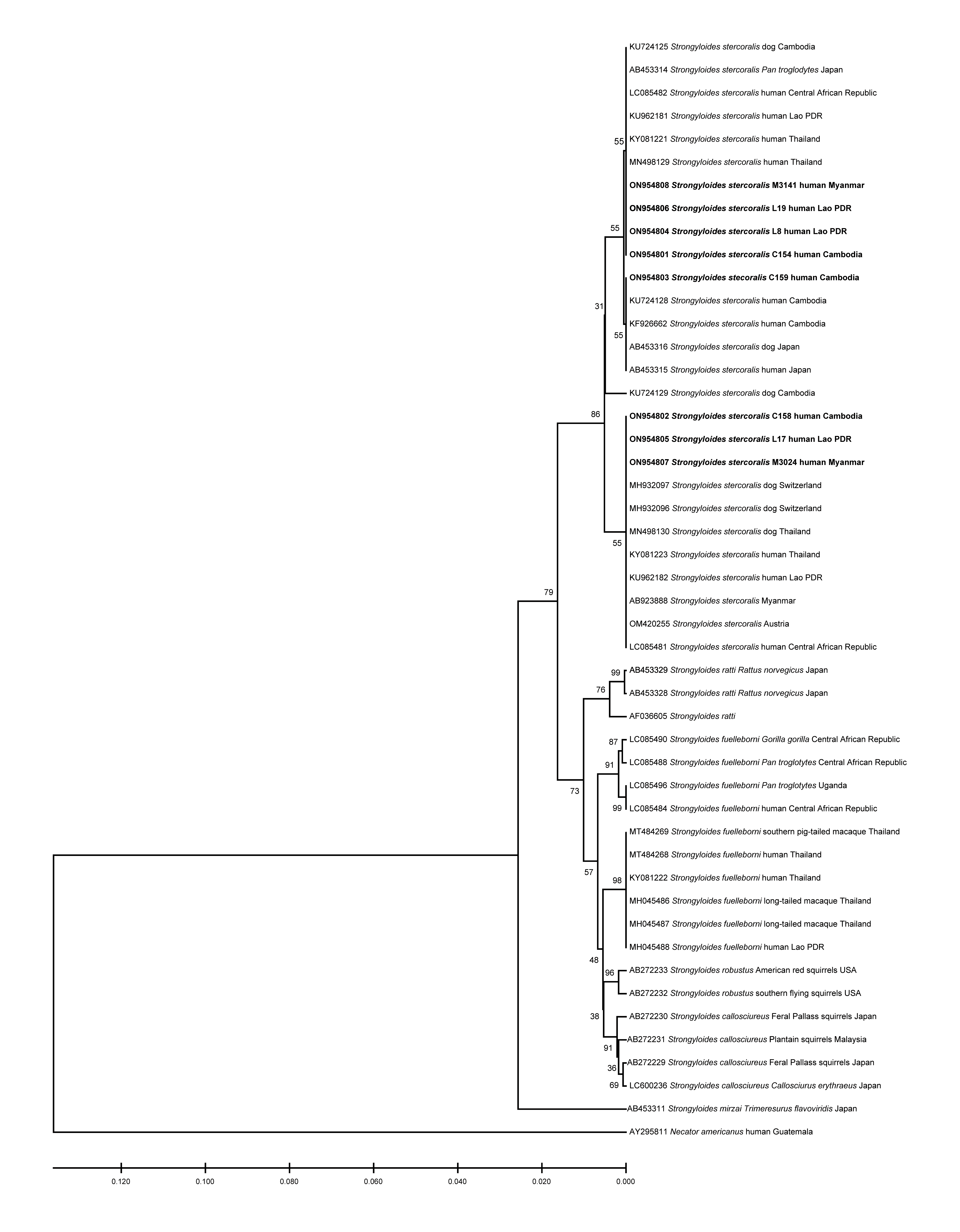

Supplement: S1 Fig — Bootstrap scores (percentages of 1000 replications) are presented for each node. The sequences of Strongyloides species obtained from GenBank database and this study (bold letters) are indicated with their accession number, hosts, and country. Necator americanus was used as an outgroup. (TIF) [file pone.0279754.s002.tif]
